# Sleep disordered breathing and its relation to stroke and pulmonary hypertension in children with sickle cell disease: a single-center cross-sectional study

**DOI:** 10.1007/s00277-023-05099-4

**Published:** 2023-01-16

**Authors:** Azza Tantawy, Nayera El-Sherif, Sara Makkeyah, Nahed Salah Eldeen, Noura Bahaa El-Din Farghal, Nanies Soliman, Fatma S. E. Ebeid

**Affiliations:** 1 Pediatric Hematology Oncology BMT, Faculty of Medicine, Ain Shams University, Cairo, 11566 Egypt; 2 Neurology Department, Faculty of Medicine, Ain Shams University, Cairo, Egypt; 3 Pediatric Cardiology, Faculty of Medicine, Ain Shams University, Cairo, Egypt

**Keywords:** Sickle cell disease, Sleep disordered breathing, Pulmonary hypertension, Stroke

## Abstract

Sleep disordered breathing (SDB) is a common underdiagnosed sequela of sickle cell disease (SCD) that has been linked to the frequency of vaso-occlusive crises. To determine the frequency of SDB in children with SCD and its association to SCD-related complications, thirty children and adolescents with SCD at their steady state underwent clinical, laboratory, and radiological assessment using transcranial duplex (TCD) and echo assessment of tricuspid regurge velocity (TRV). All participants had an overnight polysomnography after completing the modified STOP-Bang questionnaire. The mean age of the studied cohort was 10.2 years, with male: female ratio 1.7:1. Six children (20%) had high-risk for obstructive sleep apnea (OSA), while nine (30%) were at intermediate risk. Sleep apnea defined as apnea (AHI) > 1 event/hour was found among 18/30 (60%) subjects (14 males and 4 females). Children with AHI > 5 (moderate to severe OSA) had significantly higher TRV (*p* = 0.007) and left MCA flow velocity (*p* = 0.049) when compared to those with AHI < 5. Children with AHI > 5 were at higher risk of OSA according to the modified STOP-Bang questionnaire (*p* = 0.02). AHI positively correlated with TRV (*r* = 0.53, *p* = 0.003), right MCA flow velocity (*r* = 0.45, *p* = 0.013), and left MCA flow velocity (*r* = 0.55, *p* = 0.002), and negatively correlated to BMI-SDS (*r* =  − 0.48, *p* = 0.008). The high frequency of OSA in the studied cohort with SCD and its association with increasing risk of PH and TCD changes highlights the importance of early detection and management of OSA in children with SCD.

**Supplementary information:**

The online version contains supplementary material available at 10.1007/s00277-023-05099-4.

## Introduction

Sickle cell disease (SCD) is a monogenic, yet highly phenotypically variable disease with multisystem pathology with special consideration to vasculopathy as the main orchestrator of many morbidities including ischemic stroke and pulmonary hypertension [1].

One of the commonly underdiagnosed sequelae of SCD is sleep disordered breathing (SDB), including obstructive sleep apnea (OSA) and tonsillar hypertrophy. It mostly presents itself in the form of daytime sleepiness, behavioral changes, cognitive deficits, growth delays, and cardiovascular complications, and has been related to the intermittent hypoxemia, hypercapnia, and fragmented sleep associated with SCD [2]. The pathophysiologic consequences of SDB may include endothelial dysfunction with altered nitric oxide bioactivity, altered redox biology, chronic systemic inflammation, and increased expression of cell adhesion molecules [3]. Several studies demonstrated an association between nocturnal desaturations and severity of anemia [4], frequency of vaso-occlusive crises (VOC) and acute chest syndrome (ACS) [5], cardiovascular abnormalities [6], priapism [7], and neurological events [8]. Hypoxemia resulting from the recurrent apneic or hypopnic events can trigger red blood cell sickling which leads to VOC and hemolytic episodes, making both SCD and SDB share an overlapping pathophysiological process [7].

Neurological complications are among the most disabling consequences of SCD, including overt stroke, silent cerebral infarcts (SCI), and neurocognitive dysfunction [9]. The incidence of stroke and SCI is around 11%, and 37% respectively in pediatric population under 14 years of age [10][10]. In SCD, there is an increased risk of cerebral ischemia with tissue injury due to abnormal cerebral hemodynamics along with chronic anemia, and diurnal and nocturnal hypoxemia [12]. On the other hand, SDB plays an essential role in triggering these changes in children with SCD [13]. Transcranial Doppler (TCD) is a noninvasive screening modality used to assess risk of stroke in children with SCD. Those with high mean flow velocities in major brain arteries more than 170 cm/s have increased risk to stroke [14].

Pulmonary hypertension (PH) is a well-known and often fatal complication [15] with an incidence of 11–46% in children with SCD [16]. PH in SCD can be a sequela of increased pulmonary blood flow secondary to chronic anemia, chronic hemolysis, upper airway obstruction, chronic oxygen desaturation, and repeated episodes of VOCs or ACS [17]. OSA is associated with repetitive nocturnal arterial oxygen desaturation and hypercapnia, large intrathoracic negative pressure swings, and acute increases in pulmonary artery pressure (PAP). It has been also found that mild or moderate PH in the absence of any evident alternate cause can be detected in approximately 20 to 40% of individuals with OSA [18].

For the previously mentioned causes, and due to the lack of studies that correlate both cardiovascular and cerebral complication to sleep disorders in children with SCD, we designed the current study to assess the frequency of OSA in SCD in the pediatrics age group and its relation to alterations in the cerebral blood flow and pulmonary hypertension, aiming to modulate the standard of care.

## Patients and methods

### Study population

This is a single-center cross-sectional study that included thirty children and adolescents with a confirmed diagnosis of SCD aged 6 to 18 years old. They were recruited from the regular attending physicians of the Pediatric Hematology Clinic at Ain Shams University Children’s Hospital during the period from July 2019 to February 2022. All participants were at their steady state defined as “a point in time where the patient in question is not experiencing an acute painful crisis or any changes due to therapy” [19]. Children with chronic hypoxemic conditions, those receiving oxygen therapy during polysomnography, and patients receiving PH-targeted therapies were not eligible. The procedures applied in this study were approved by the Institutional Review Board of the Children’s Hospital of Ain Shams University and the Research Ethics Committee of Human Experimentation at Ain Shams University (M.D156/2019) and are in accordance with the ethical principles for medical research as in Helsinki Declaration of 2008. An informed consent was obtained from all legal guardians of the participants while an assent form was obtained from participant whenever applicable.

### Methods and study tools

All the participants were thoroughly evaluated with special emphasis on anthropometric measurements (weight, height, body mass index, and neck circumference), neurological and cardiac examination, and SCD-related complications. The following equation was used to calculate the transfusion index: the volume of transfused packed red cells in milliliter per kilogram body weight per year (expressed as the mean value in the last 2 years). Eighteen children with SCD (60%) received chelation therapy; twelve of them received oral deferasirox in single daily dose of 20–40 mg/kg/day while four patients received deferoxamine (DFO) infused subcutaneously in a dose 30–45 mg/kg/day given 5 days/week and only two patients received oral deferiprone (DFP) in a daily dose of 50–100 mg/kg/day. Twenty-nine patients (96.7%) received oral hydroxyurea therapy at a dose of 20 mg/kg/day with escalation to a maximum tolerated dose according to the safety and response as illustrated in Table 1. Examination of ear, nose, and throat (ENT) was performed to all the enrolled children to rule out peripheral causes of apnea.

**Table 1:** Clinical and laboratory characteristics of study participants

Variable	Number = 30
Age (years); mean ± SD	10.22 ± 3.28
Gender *n* (%)	
Female	11 (36.7%)
Male	19 (63.3%)
Weight SDS; median (IQR)	− 0.5 (− 2.26–0.5)
Height SDS; median (IQR)	− 2 (− 2.72 to − 1.2)
BMI SDS; median (IQR)	− 0.51 (− 1.85–0.8)
Neck circumference n (%)	
< 95th centile	28 (93.3%)
≥ 95th centile	2 (6.7%)
CBC and Hb analysis	
Hemoglobin (g/dl); mean ± SD	7.12 ± 1.46)
Hematocrit (%); mean ± SD	22.56 ± 3.48
Total leucocyte count (× 10^*3^/ul); mean ± SD	14.93 ± 8.95
Platelet count (× 10^*3^/ul); mean ± SD	400.00 ± 181.94
Hb S (%); mean ± SD	65.27 ± 13.16
Hb F (%); median (IQR)	10 (5.8–15)
Comorbidities; *n* (%)	
Sickle crises > 3/y	12 (40.0%)
Left ventricular dilatation	1 (3.3%)
Abnormal finding on MRI brain	7 (23.3%)
Nocturnal enuresis	10 (33.3%)
History of overt stroke	6 (20.0%)
Blood transfusion	
Occasional transfusion	18 (60.0%)
Simple transfusion	7 (23.3%)
Exchange transfusion	5 (16.7%)
Hydroxyurea and chelation therapy	
Hydroxyurea	29 (96.7%)
Chelation	
No chelation	12 (40.0%)
Deferasirox	12 (40.0%)
Deferoxamine	4 (13.3%)
Deferiprone	2 (6.7%)

*SD*, standard deviation; *IQR*, interquartile range; *Hb*, hemoglobin; *N*, number

Laboratory investigations included complete blood count (CBC) using Sysmex XT-1800i (Sysmex, Kobe, Japan), hemoglobin analysis by HPLC using D-10 (Bio-Rad, Marnes La Coquette, France), and markers of hemolysis (lactate dehydrogenase and indirect bilirubin) using Cobas Integra 800 (Roche Diagnostics, Mannheim, Germany) and serum ferritin level was measured on Immulite 1000 analyzer (Siemens Healthcare Diagnostics, Marburg, Germany).

#### Modified STOP-Bang questionnaire

This is a modified version of a commonly used adult clinical prediction tool for stratifying the risk of OSA [20] that has been tailored to the pediatric population. Patients were stratified for OSA risk according to their modified STOP-Bang scores as illustrated in the supplement file [21].

The question concerning the academic problems in the adult version of the questionnaire “age greater than 50” was replaced by a question for the care givers “Does your child have learning problems?” in order to emphasize the correlation between SDB, and neurocognitive functions and school performance in children [22]. We used a verified Arabic version of STOP-Bang questionnaire to match with our Arabic-speaking patients [23].

#### Transcranial Doppler

Mean blood flow velocities of the middle cerebral artery (MCA) on both sides of the brain were examined to evaluate the risk of stroke [14]. The classification was established based on the highest time averaged maximum mean velocity (TAMMV) measured in the MCA during the examination. The TAMMV in each vessel was classified using the criteria identified in the stroke Prevention Trial in Sickle Cell Anemia (STOP trial) [24].

#### Echocardiography

Pulmonary pressure was estimated for all children using transthoracic Doppler echo cardiography by measuring the tricuspid regurgitant jet velocity (TRV). Abnormal TRV ≥ 2.5 m/s is a reliable predictor of elevated pulmonary artery pressures [25].

#### Polysomnography

This is an overnight multi-parametric sleep study test which was performed using a sleep monitor (Natus Nicolet EEG n32, USA) in the sleep laboratory of the neurology department of Ain Shams University Hospitals. It records snoring, abdominal and thoracic respiratory effort, nasal cannula flow, body position, oxygen saturation, heart rate, peripheral capillary oxygen saturation, and one channel EEG. Oxygen desaturations were classified as mild (SpO2 of 90–94%), moderate (75–89%), and severe (less than 75%). Scoring of sleep was performed by a single registered polysomnographic technologist using the standard criteria [26]. Apneas were scored when there is a decrease in the amplitude of the thermistor airflow ≥ 25% of the pre-event baseline and lasted for more than 6 s or 2 breath cycles*.* Hypopneas were designated if the amplitude of any respiratory signal decreased below 70% of the baseline amplitude, was associated with ≥ 3% oxygen desaturation, and the thermistor signal did not meet the criterion for apnea [22]. Apnea hypopnea index (AHI) was defined as the number of apneas and hypopneas per hour of total sleep time. For data analysis, AHI more than 1 was considered to indicate OSA, with cut-off points of 1, 5, and 10 events/hour have been suggested to indicate mild, moderate, and severe levels respectively [27].

#### Statistical analysis

Statistical analysis was performed through SPSS software version 27 (IBM SPSS Statistics, IBM Corporation, Chicago, IL, USA). Kolmogrov–Smirnov test was used to examine the normal distribution of variables. Quantitative variables were described in the form of mean and standard deviation or median and interquartile range (IQR: 25th–75th percentiles). Qualitative variables were described as number and percent. To compare parametric quantitative variables between two groups, Student’s *t*-test was applied. For comparison of non-parametric quantitative variables between two groups, Mann–Whitney test was used. Qualitative variables were compared using chi-square (X^2^) test or Fischer’s exact test when frequencies were below five. Pearson’s correlation coefficients were used to assess the association between two normally distributed variables. When a variable was not normally distributed, a Spearman’s correlation test was performed. A one-way ANOVA “analysis of variance” was used to compare the means of three or more independent groups to determine if there is a statistically significant difference between the corresponding population means. A *p* value < 0.05 was considered significant in all analyses.

## Results

The study subjects consisted of 30 children with established diagnosis of SCD (19 males and 11 females with ratio 1.7:1 ratio, mean age: 10.22 ± 3.28 years). Patients’ characteristics and laboratory investigations are presented in Table 1. Ten patients (33.3%) suffered from nocturnal enuresis, one had cardiac complication in the form of left ventricular dilatation, and six (20%) patients had previous stroke. Twelve participants (40%) received regular transfusion either in the form of simple transfusion (7 patients) or automated red cell exchange (5 patients; all with previous stroke). Eighteen patients (60%) were on chelation therapy during the study period.

**Fig. 1:**
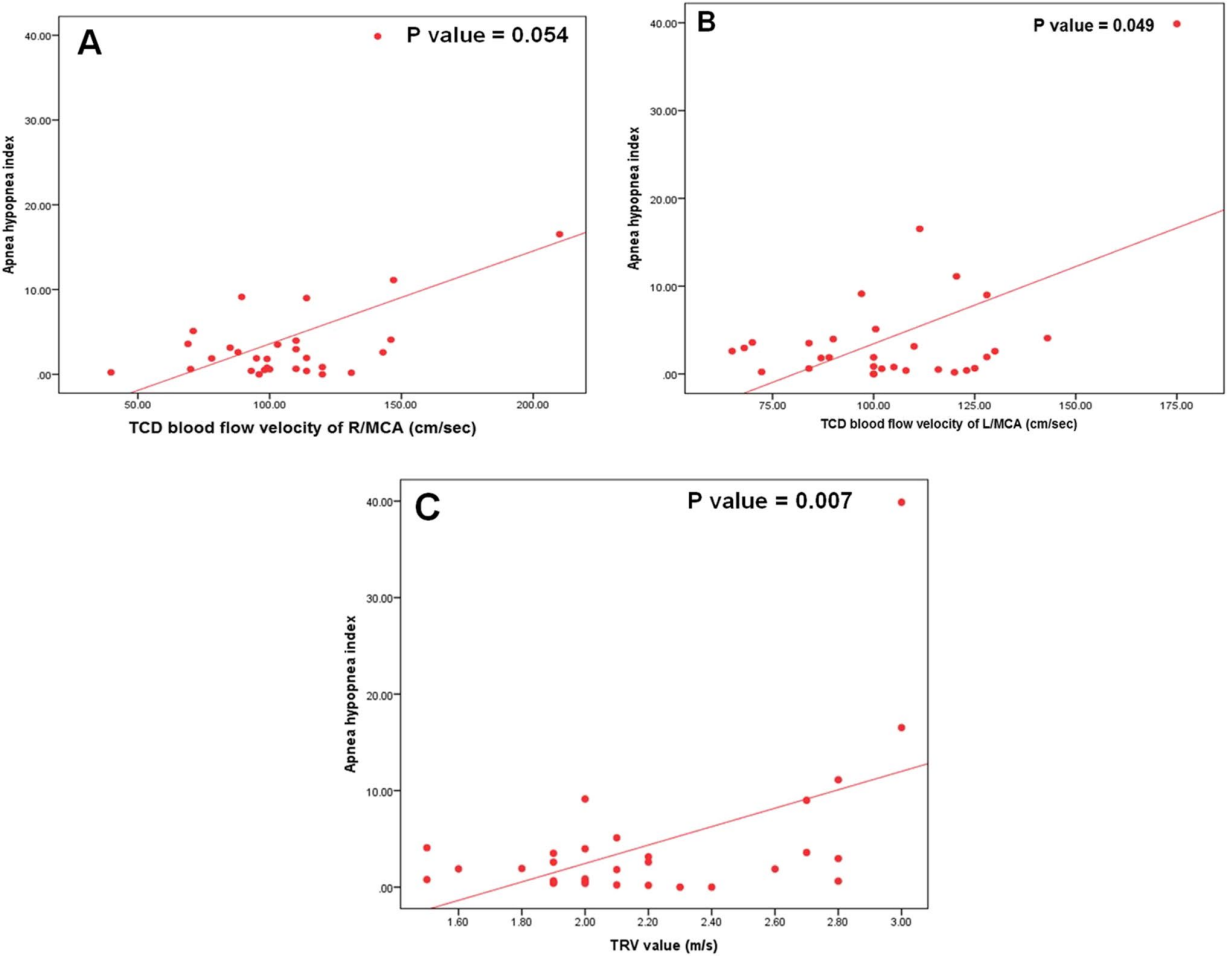
Correlation between apnea hypopnea index (events per hour) and different parameters:A Transcranial Doppler (TCD) blood flow velocity of right middle cerebral artery (R/MCA) (cm/sec). B TCD blood flow velocity of left MCA(L/MCA) (cm/sec). C Tricuspid regurge velocity (TRV) value (m/s)

To determine the effect of the age on AHI, we compared two different age groups: 6–13 years (*n* = 22) and 13–18 years (*n* = 8). Younger children (6–13 years) had significantly lower AHI than those aged 13–18 years (Fig. 1), while there was no significant difference in the frequency of normal, mild, moderate, and severe AHI between the two age groups (*p* = 0.182).

The mean right and left MCA flow velocity were 106.77 ± 31.42 cm/s and 105.06 ± 23.89 cm/s respectively. Four out of the thirty studied participants (13.3%) had abnormal low TCD flow velocity, one patient (3.3%) had conditional TCD flow velocity, and another patient (3.3%) showed high TCD flow velocity. The mean TRV was 2.2 m/s among the studied group, of which eight (26.7%) subjects showed abnormal high TRV as illustrated in Table 2.

**Table 2:** Echocardiography and transcranial Doppler assessment of the studied group

Variable	No. = 30
Echocardiography	
TRV value (m/s); mean ± SD	2.20 ± 0.43
Abnormal TRV > 2.5 m/s; *n* (%)	8 (26.7%)
Transcranial duplex (TCD)	
Right MCA; mean ± SD	106.8 ± 31.4
Left MCA; mean ± SD	105.1 ± 23.9
TCD category; *n* (%):	
Normal	24 (80.0%)
Conditional	1 (3.3%)
Abnormal very low	4 (13.3%)
Abnormal high	1 (3.3%)

*TRV*, tricuspid regurge velocity; *TCD*, transcranial Doppler; *SD*, standard deviation; *N*, number

**Fig. 2:**
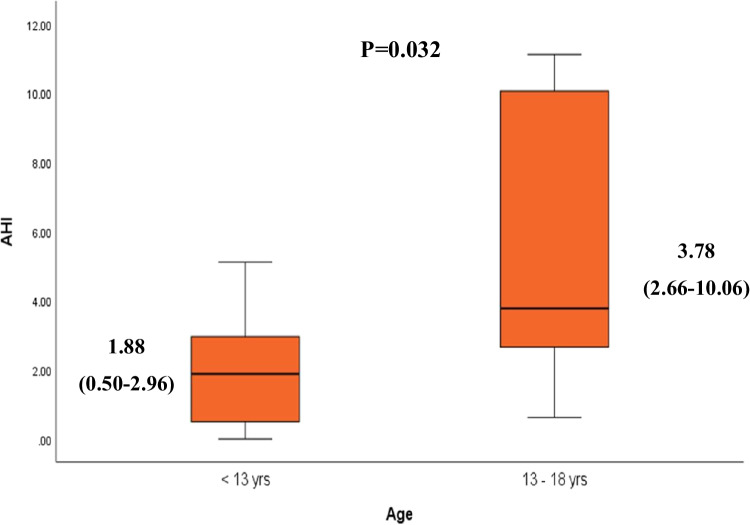
Comparison of median AHI between two different age groups

According to the modified STOP-Bang questionnaire, nine patients (30%) were classified as intermediate risk, and six patients (20%) as high risk for sleep apnea. Analysis of different variables among the three risk groups (low, intermediate, and high) revealed that all patients with neck circumference > 95th percentile were among the high-risk group, while those in the low and intermediate risk groups tend to have normal neck circumference. There was a significant lower TRV value and lower frequency of abnormal TRV in low-risk patients (*p* < 0.0001, 0.045 respectively). TCD has a predictive contribution to OSA risk; all patients with low risk had normal TCD in comparison to 55.6% and 66.7% with intermediate and high risk (*p* = 0.049). Correlation between the modified STOP-bang questionnaire risk and different variables showed positive correlation with TRV value (m/s) (*r* = 0.660, *p* = 0.000) and total hypopnea (*r* = 0.423, *p* = 0.020), while it showed negative correlation with total sleep time (*r* =  − 0.473, *p* = 0.008) and baseline pre-sleep O_2_ saturation (*r* =  − 0.364, *p* = 0.048) (Fig. 2).

Characteristics of polysomnography for the studied group are presented in Table 3. The mean total sleep time was 4.12 ± 1.5 h with sleep efficiency of 75.87% ± 22. One patient had total sleep duration of 00:39:00 h causing skewness of data; with exclusion of data of this patient, the median of the total sleep hours was 4.5 ranging 1.54–6.44 h. The median (IQR) AHI was 1.91 events/hour (0.59–3.97). Sleep apnea, defined as AHI > 1 event/hour, was found in 18/30 (60%) of patients (14 males and 4 females).

**Table 3:** Modified STOP-Bang questionnaire and polysomnography findings among the study participants

Variable	*N* = 30
Modified STOP-Bang questionnaire risk	
Low	15 (50.0%)
Intermediate	9 (30.0%)
High	6 (20.0%)
Polysomnography	
Total sleep time (hours); mean ± SD	4.12 ± 1.50
Sleep onset latency (hours); median (IQR)	0.25 (0.17–0.35)
Sleep efficiency (%); mean ± SD	75.87 ± 22.05
REM duration (hours); median (IQR)	0 (0–0.08)
REM latency (hours); mean ± SD	2.04 ± 0.66
Respiratory sleep apnea	
AHI; median (IQR)	1.91 (0.59–3.97)
AHI category, *n* (%)	
Normal (0–1)	12 (40.0%)
Mild (> 1–5)	12 (40.0%)
Moderate (> 5–10)	3 (10.0%)
Severe (> 10)	3 (10.0%)
Hypopnea average duration (seconds); median (IQR)	17.45 (13.9–30)
No. of hypopneas; median (IQR)	5.5 (2–12)
No. of apneas; median (IQR)	1 (0–6)
Sleep summery	
Total stage shift (*n*); median (IQR)	9.5 (7–17)
Total awakening (*n*); median (IQR)	2 (1–3)
Respiratory sleep stages (apneas/hypopneas)	
Stage N1%	
Apnea (*n*); median (IQR)	0 (0–0)
Hypopnea (*n*); median (IQR)	0 (0–4)
Stage N2%	
Apnea (*n*); median (IQR)	0 (0–3)
Hypopnea (*n*); median (IQR)	1.5 (0–6)
Stage N3%	
Apnea (*n*); median (IQR)	0 (0–2)
Hypopnea (*n*); median (IQR)	1 (0–5)
REM sleep	
Apnea (*n*); median (IQR)	0 (0–0)
Hypopnea (*n*); median (IQR)	0 (0–0)
Total number	
Apnea (*n*); median (IQR)	1 (0–6)
Hypopnea (*n*); median (IQR)	4.5 (1–12)
Oxygen saturation	
Baseline O2 saturation (%); mean ± SD	96.13 ± 3.67
Minimum saturation (%); mean ± SD	66.70 ± 12.57
Maximum saturation (%); mean ± SD	99.50 ± 0.97

*RDI*, respiratory disturbance index

Patients with AHI > 5 (moderate to severe OSA) had significantly higher TRV and left MCA flow velocity when compared to those with AHI < 5 as depicted in Table 4. Other variables including BMI-SDS (*p* = 0.07), presence of cardiac dysfunction (*p* = 0.26), history of stroke (*p* = 0.17), presence of nocturnal enuresis (*p* = 0.33), WBCs count (*p* = 0.23), hemoglobin (*p* = 0.087), hematocrit (*p* = 0.264), platelet count (*p* = 0.730), LDH (*p* = 1.00), hemoglobin S (*p* = 0.158), and hemoglobin F (*p* = 0.588) were comparable between both groups (Table 3).

**Table 4:** Comparison of clinical data, modified STOP-Bang questionnaire, and radiological findings between patients with low risk and moderate to severe risk OSA

	AHI < 5 No. = 24	AHI > 5 No. = 6	*P*-value
Age (years); mean ± SD (range)	10.04 ± 3.06 (6–16)	10.92 ± 4.34 (6.5–17)	0.569
Gender Female Male	10 (41.7%) 14 (58.3%)	1 (16.7%) 5 (83.3%)	0.256
Nocturnal enuresis	7 (29.2%)	3 (50.0%)	0.333
Previous stroke	6 (25.0%)	0 (0.0%)	0.171
BMI (SDS); median (IQR)	− 0.27 (− 0.99–0.85)	− 1.93 (− 2.9 to − 1.29)	0.066
Neck circumference < 95th centile > 95th centile	22 (91.7%) 2 (8.3%)	6 (100%) 0 (0%)	0.464
Modified STOP-Bang questionnaire risk; ***n*** (%)			
Low	15 (62.5%)	0 (0%)	0.020
Intermediate	5 (20.8%)	4 (66.7%)	
High	4 (16.7%)	2 (33.3%)	
Echocardiography			
TRV value (m/s); mean ± SD	2.10 ± 0.36	2.60 ± 0.44	0.007
TRV category; *n* (%)			
Normal	20 (83.3%)	2 (33.3%)	0.013
Abnormal	4 (16.7%)	4 (66.7%)	
Transcranial duplex (mean ± SD)			
Right middle cerebral artery	101.28 ± 23.61	128.73 ± 49.37	0.054
Left middle cerebral artery	100.80 ± 21.20	122.07 ± 28.45	0.049
TCD Category; *n* (%)			
Normal	20 (83.3%)	4 (66.7%)	0.027
Conditional	0 (0.0%)	1 (16.7%)	
Abnormal very low	4 (16.7%)	0 (0.0%)	
Abnormal high	0 (0.0%)	1 (16.7%)	

*SD*, standard deviation; *IQR*, interquartile range; *N*, number; *RDI*, respiratory disturbance index; *TRV*, tricuspid regurge velocity; *TCD*, transcranial doppler

**Table 5:** Relation of minimum oxygen saturation with AMH, TRV, TCD, and M. STOP bang Q. risk

		Minimum O2 saturation		*P*-value
		Mean ± SD	Range	
Apnea hypopnea index (AHI)	Normal	69.45 ± 15.02	50–84	0.575•
	Mild	65.25 ± 12.45	51–83	
	Moderate	66.67 ± 2.52	64–69	
	Severe	58.00 ± 7.00	53–66	
Tricuspid regurgitant jet velocity (TRV)	Normal	69.45 ± 12.53	50–84	0.022^≠^
	Abnormal	59.13 ± 9.70	50–77	
Transcranial duplex (TCD)	Normal	66.83 ± 12.38	50–84	0.436•
	Conditional	53.00 ± 0.00	53–53	
	Abnormal very low	72.25 ± 14.27	51–81	
	Abnormal high	55.00 ± 0.00	55–55	
Modified STOP-Bang questionnaire RISK	Low	70.37 ± 12.89	50–84	0.215•
	Intermediate	61.73 ± 11.43	50–81	
	High	65.33 ± 12.01	53–77	

•, one-way ANOVA; ≠ , independent *t*-test

**Fig. 3:**
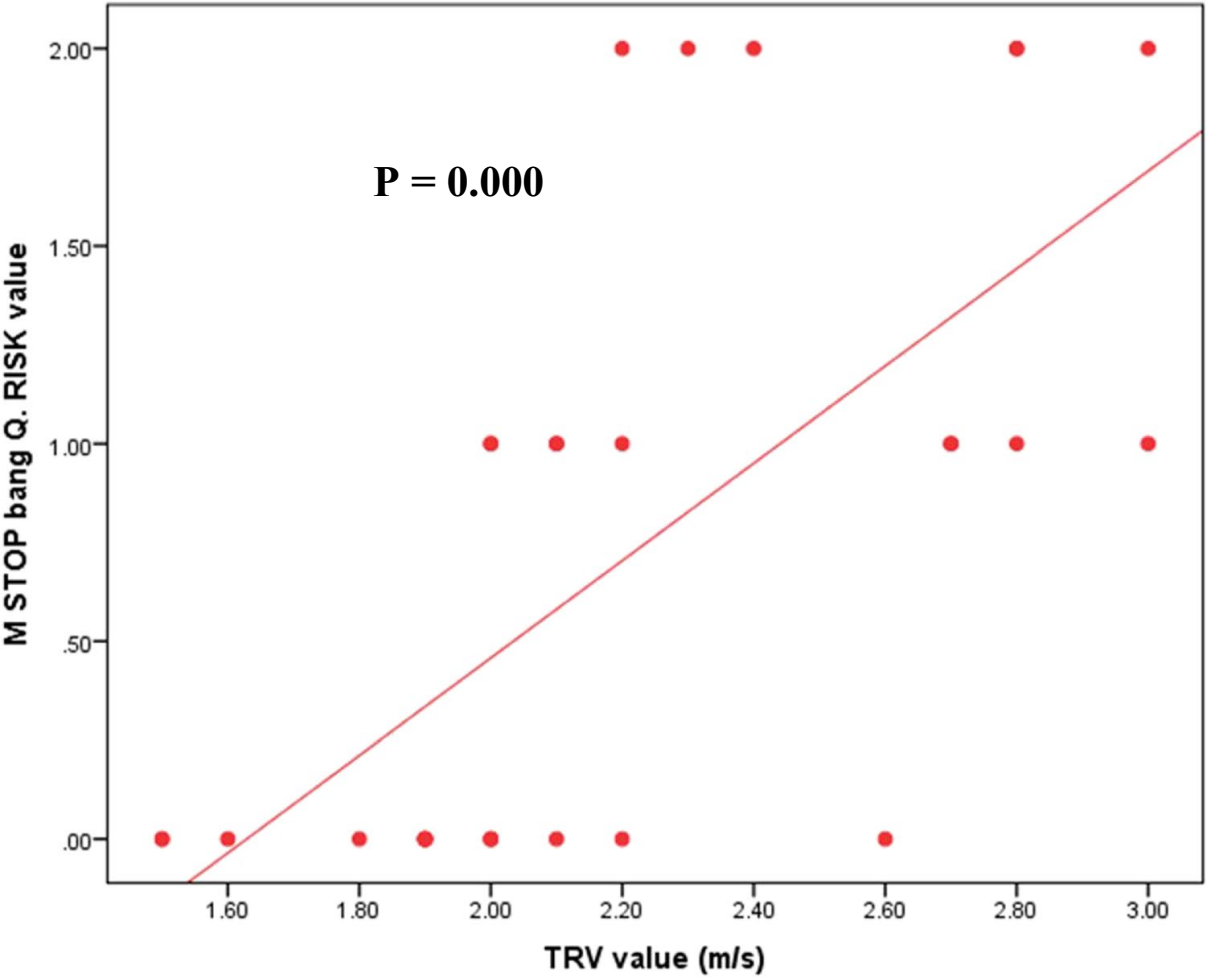
Correlation between modified stop bang questionnaire risk and tricuspid regurgitation velocity of the studied patients

Patients with AHI > 5 were at higher risk of OSA according to the modified STOP-Bang questionnaire (*p* = 0.02). AHI was positively correlated with TRV (*r* = 0.53, *p* = 0.003), right MCA flow velocity (*r* = 0.45, *p* = 0.013), and left MCA flow velocity (*r* = 0.55, *p* = 0.002), while it showed negative correlation with BMI-SDS (*r* =  − 0.48, *p* = 0.008) (Fig. 3). AHI was positively correlated with exchange frequency/month (*r* = 0.938, *p* = 0.018), total apnea (*r* = 0.816, *p* = 0.000), and total hypopnea (*r* = 0.933, *p* = 0.000) and showed non-significant correlation to age, HbF%, HbS%, and HU intake (Table 5).

Four patients had adeno-tonsillar hypertrophy (ATH), two of them underwent adenoido-tonsillectomy. To assess the effect of ATH on sleep and OSA, we compared the two groups (4 children with ATH and 26 without ATH) and found no significant difference as regards AHI value, TRV value, and TRV category (*p* = 0.051, 0.224, 0.257 respectively).

Considerable hypoxemia is defined as a mean minimum oxygen saturation of 75%. We analyzed the relation between the mean minimum oxygen saturation and different parameters. The results showed no correlation between the mean minimum oxygen saturation and the AHI (*r* =  − 0.275, *p* = 0.149), TRV value (*r* =  − 0.242, *p* = 0.197), or TCD (*r* = 0.12, *p* = 0.526). By performing analysis between mean minimum oxygen saturation and categories of the above-mentioned parameters, significant lower value of mean minimum oxygen saturation and those with abnormal TRV (*p* = 0.022) was observed.

## Discussion

Hypoxemia-related vasculopathy is a common endpoint leading to morbidity in SCD. The vulnerability of the abnormal hemoglobin S to polymerization and sickling during hypoxemia is the main pathophysiology that leads to vaso-occlusive crises and different morbidities [1]. In addition, children and adolescents with SCD are not only at an increased risk of SDB [28], but SDB itself predisposes to nocturnal hypoxemia. For this reason, a common pathogenesis exists between SCD and SDB related to ischemia, hypoxemia, reperfusion injury, and endothelial dysfunction [29].

Forty percent of our study cohort suffered from SDB, which is considered a relatively common occurrence. Traditionally, risk factors for OSA in children included obesity [21], adenotonsillar hypertrophy (ATH) [30], airway narrowing, and craniofacial abnormalities. It has been shown that each increment in BMI above the 50th percentile is associated with around a 10% increased risk for OSA [31]; however, this was not demonstrated in our studied group. This could be explained by the possible impact of other risk factors on OSA, like ATH and anatomic changes in the upper airway secondary to bone marrow hyperplasia; as an evidence of our results, four out of the six patients with moderate to severe OSA had history of ATH. Although the cause of ATH in SCD is not well established, it could be related to the chronic inflammatory state, recurrent infections due to asplenia and defective opsonization, or reticuloendothelial hyperplasia [32].

In our cohort, four patients had ATH who was suffering from poor sleep quality had higher risk of OSA according to the modified STOP-Bang questionnaire and higher AHI. Two out of the four patients underwent adenotonsillectomy with marked improvement of OSA symptoms.

The influence of age as an adverse sequel was more apparent with maturity as there was a significant higher value of AHI in older patients. This cofounder could be explained by increasing risk of ATH in older patients. A previous study did not observe a significant increase in the prevalence of ATH among children with SCD, aged 2 to 6 years [33]. This fact makes it possible to speculate that the growth of the tonsils in children with SCD can occur even after preschool age. Some authors believe that this hypertrophy may occur due to self-splenectomy, which would also be in accordance with a higher incidence of ATH in adolescents than in infants with SCD [34].

The STOP-Bang questionnaire that was originally developed to predict pre-operative OSA risk [35] appears to be a promising screening tool for OSA in adult patients at risk for PH [36]. While in pediatrics age group, the modified STOP-Bang questionnaire was evaluated in previous studies and proofed to have excellent sensitivity, but modest specificity in detecting OSA [37]. By using the modified STOP-Bang questionnaire, 50% of our studied sample had intermediate to high risk of OSA. Moreover, higher scores on the modified STOP-Bang questionnaire were associated with SDB found on overnight PSG.

The modified STOP-Bang questionnaire may represent an alternative tool to the expensive pediatric polysomnography which is not commonly available in areas without specialized pediatric centers. It has been shown to stratify the risk of OSA with a negative predictive value of 93% and 96% for moderate and sever OSA respectively [38]. It also could be a valuable non-lengthy clinical prediction tool which is easily administered in a busy “real-world” care setting used to determine the need for PSG in children and adolescents with SCD.

By using this questionnaire, a significant difference between different scores and TCD results was found in the studied population. As all SCD patients with low risk to OSA showed normal TCD, 55.6% in the intermediate risk group and 66.7% in the high-risk group. This agrees with castello-branco et al., who found a significant negative correlation between the risk of OSA calculated by STOP-Bang and the breath-holding index calculated using transcranial duplex which assess cerebral vasoreactivity [39].

The TCD is a non-invasive, inexpensive, and safe technique for assessment of intracerebral blood flow [40]. It can detect overt stroke and predict early ischaemic recurrence in patients with stroke or TIA of arterial origin [41], and it can also efficiently detect SCD patients at risk of stroke [42]. The STOP-I study clearly demonstrated that an abnormal TCD was predictive of stroke risk (10% per year) in SCA patients [42]. Although stroke can occur at any age, the most vulnerable group for ischemic stroke is between the age of 2 and 20 years [10]. Stenotic lesions involve primarily large vessels in the intracranial internal carotid, middle, and anterior cerebral circulation and can progress for months and even years before symptoms develop [43]. These changes can be detected by TCD and detect patients with cerebral vasculopathy [42].

Cerebral infarction is a multifactorial devastating complication in SCD due to narrowing and occlusion of the intracranial arteries, anemia, hypoxemia, impaired cerebral autoregulation, increased blood viscosity, lung disease, and acute medical events resulting in reduced baseline hemoglobin [44]. Twenty percent of the studied patients showed abnormal TCD findings and higher TCD velocities; this was demonstrated in patients with moderate to severe OSA (AHI > 5) using polysomnography. This could be explained by the recurrent hypoxemia from OSA which contributes to vessel abnormalities and vaso-occlusion in small cerebral vessels [45]. Various studies have documented impaired cerebral perfusion during obstructive apneas events [46, 47]. Hill et al. found significant elevation in TCD velocities in 31 healthy children with mild SDB (AHI < 5) compared to 17 age-similar control patients [48].

Echocardiographic estimation of pulmonary artery pressure (PAP) by measuring the TRV is a validated useful screening method for PH in adult patients with SCD [49]. A jet velocity ≥ 2.5 m/s, which corresponds to a systolic PAP ≥ 30 mmHg, was used to define elevated PAP in adults with SCD and the prevalence is about 30% [50]. To determine whether PH can be present in OSA in the absence of hypoxic lung disease, Sajkov et al. studied 32 patients with OSA and found PH (estimated mean PAP ≥ 20 mmHg) in 34% of the subjects [51]. Hetzel et al. [50] studied 49 consecutive patients with OSA with normal pulmonary function testing and found six patients (12%) with resting PH using a mean PAP > 20 mmHg. Ulrich et al. observed sleep apnea, mainly central sleep apnea (CSA), in 17 (45%)out of 38 patients with different etiologies of PH [51].

In our study, the mean TRV was 2.20 ± 0.43 m/s; moreover, 8/30 (26.7%) of the studied pediatric sample had abnormal TRV > 2.5 m/s showing significant positive relation between OSA risk (using the modified STOP bang questionnaire and polysomnography) and TRV. These results from the pediatrics age group support the data from previous work in the adult age group [51] which found an increasing proportion of cardiovascular events including PH across the low, intermediate, and high OSA risk groups classified by the STOP-Bang questionnaire. Pathophysiological mechanism can include the chronic vasculopathic and hemolytic nature of SCD [52] along with the repetitive nocturnal desaturations and pulmonary arteriolar remodeling and hyper-reactivity to hypoxia in OSA [18].

In our study, the minimal oxygen saturation was measured instead of nocturnal hypoxia, and we found that hypoxia is one of drivers of adverse cardiovascular findings especially as there was significant lower value of mean minimum oxygen saturation in those with abnormal TRV.

In a retrospective chart review of 20 children with SCD who performed polysomnography and echocardiogram within a narrow time interval, 25% had nocturnal hypoxia and 40% predominantly male had evidence of PH. Children with nocturnal hypoxia had significant worse baseline hypoxemia and higher TRV. Although the severity of nocturnal hypoxia was influenced by OSA, PH was not associated with OSA [53].

Limitations to our work include the cross-sectional nature of the study and the use of a convenient but small sample size. It is possible that a larger prospective study would have resulted in more significant associations and would have allowed a multivariate analysis of potential risk factors for SDB.

Another limitation in our work was the relatively short mean total sleep time in the studied cohort which was around 4 h. Performing polysomnography was challenging in the studied cohort, as children have a limited ability to cooperate with the setup, have trouble sleeping in a strange environment, could not accommodate sleeping long periods, and had frequent arousals.

## Conclusion

The high frequency of OSA in the studied pediatric cohort with SCD and its association with increasing risk of PH and TCD changes emphasizes the importance of early detection and management of OSA in children with SCD using noninvasive easily implemented screening tools.

## Supplementary information

Below is the link to the electronic supplementary material.

Supplementary file1 (DOCX 12 KB)

## Data Availability

All authors are sure that all data and materials as well as software application or custom code support their published claims and comply with field standards.

## Code availability

Non-applicable.
