# Supplementary material for: Sleep disordered breathing and its relation to stroke and pulmonary hypertension in children with sickle cell disease: a single-center cross-sectional study
Source: Ann Hematol. 2023 Jan 16;102(2):271–81. doi: 10.1007/s00277-023-05099-4 (PMC9889484; doi:10.1007/s00277-023-05099-4)
Supplement: Supplementary file 1 — Supplementary file1 (DOCX 12 KB) [file 277_2023_5099_MOESM1_ESM.docx]

Supplementary file:

The questionnaire included the following questions: Snoring (How often does your child snore loudly?), Tired (Is your child sleepy during the daytime?), Observed apnea (Does your child stop breathing during sleep?), systolic or diastolic blood pressure greater than or equal to 95^th^ percentile for height and age, BMI greater than 95^th^ percentile for age, Academic problems (Does your child have learning problems?), Neck circumference greater than 95^th^ percentile for age, and male Gender. Answers to the questions had choices that included—don’t know, never, rarely, occasionally, frequently or almost always.

The responses were collapsed into a positive or negative response in the following manner. Frequently or almost always was considered as a positive response and responses of don’t know, never, rarely, or occasionally were all considered as a negative response. The number of positive responses was calculated as the subject’s modified STOP-Bang score. The total score ranges from 0 to 8. Patients with a score of 0 to 2 were classified as low risk for obstructive sleep apnea, whereas those with a score of 5 to 8 were classified as high-risk. In patients whose modified STOP-Bang scores were in the midrange (3 or 4), other further criteria were applied for classification; a modified STOP-Bang score of ≥ 2 plus a BMI > 35 kg/m^2^ or in male gender or neck circumference above 95^th^ percentile would classify that patient at high-risk for OSA. In this way, patients can be stratified for OSA risk according to their modified STOP-Bang scores. [21]
